# Neurotoxicological Effects of Some Mycotoxins on Humans Health and Methods of Neuroprotection

**DOI:** 10.3390/toxins17010024

**Published:** 2025-01-06

**Authors:** Aleksandra Kuć-Szymanek, Daria Kubik-Machura, Klaudia Kościelecka, Tomasz Męcik-Kronenberg, Lidia Radko

**Affiliations:** 1 Faculty of Medical and Health Sciences, University in Siedlce, Stanisława Konarskiego St. 2, 08-110 Siedlce, Poland; ola.kuc98@gmail.com; 2 Provincial Specialist Hospital No. 5 St. Barbara in Sosnowiec, Trauma Center, Plac Medyków St. 1, 41-200 Sosnowiec, Poland; daria.kubik64@gmail.com; 3 Municipal Hospital in Zabrze, Zamkowa St. 4, 41-803 Zabrze, Poland; klaudia.koscielecka@gmail.com; 4 Department of Pathomorphology, Faculty of Medical Sciences in Zabrze, 3 Maja St. 13, 41-800 Zabrze, Poland; patolog@interia.pl; 5 Collegium Medicum im. Dr. Władysław Biegański, Jan Długosz University, Wahington St. 4/8, 42-200 Czestochowa, Poland; 6 Department of Preclinical Sciences and Infectious Diseases, Faculty of Veterinary Medicine and Animal Sciences, Poznan University of Life Sciences, Wolynska St. 35, 60-637 Poznan, Poland

**Keywords:** neurotoxicity, mycotoxins, *Fusarium* spp., *Penicillium* spp., *Aspergillus* spp. protection

## Abstract

Food contamination with mycotoxin-producing fungi increases the risk of many diseases, including neurological diseases closely related to the neurotoxicity of these toxins. Based on the latest literature data, we presented the association of common *Fusarium* mycotoxins with neurological diseases. Articles from 2001 to 2024 were analyzed. The mechanisms underlying the neurotoxicity of the described mycotoxins were presented. They are mainly related to the increase in oxidative stress in neuronal cells, which leads to higher levels of pro-inflammatory cytokines as IL-1β, IL-6 and TNF-α, enzymatic activity as GST, GPx, CAT and SOD and neurotransmitter dysfunction (5-HT, serotonin, dopamine and GABA). At the end of the article, based on the literature data, we attempted to present ways to mitigate mycotoxin neurotoxicity using mainly natural substances of plant origin. The data in this review focus on the *Fusarium* mycotoxins most frequently found in food and will be useful as comparative information for future studies. It is important to conduct further studies to mitigate the neurotoxic effects of *Fusarium* mycotoxins in order to reduce the development of diseases of the nervous system.

## 1. Introduction

Mycotoxins are fungal metabolites that cause harmful effects in humans and animals, produced mainly by mold fungi of the genera *Aspergillus* spp., *Penicillium* spp. and *Fusarium* spp. (Table 1). Important problems include their toxicity, high frequency of occurrence in food, long exposure of the body to mycotoxins and high stability [1,2]. The increase in crop contamination with mycotoxins is related to climate change, changes in the natural environment and inappropriate harvesting, storage and processing [2]. According to official statistics, more than 25% of global crops are contaminated with mycotoxins [3]. The alimentary route is the main route of exposure to mycotoxins and the source is food and water. The aerogenic and dermal routes of exposure are less important. Spontaneous *Fusarium* mycotoxicosis outbreaks have been reported in Europe, Asia, Africa, New Zealand and South America. In addition, chronic exposure to these mycotoxins is regularly monitored by conducting official control tests of food due to their global occurrence [4]. Mycotoxins produced by *Penicillium* spp., i.e., ochratoxin A (OTA) and patulin, found in feed, are regulated in many countries. However, in terms of human health, they are rarely associated as human pathogens, because they hardly grow at 37 °C. One of the most potent mycotoxins is aflatoxin (AF). AFs are produced by fungi belonging mainly to the genus *Aspergillus* spp. The toxic effect of these compounds leads to disturbances in the metabolism of fats, proteins and carbohydrates as well as the synthesis of nucleic acids, leading to the development of cancer, e.g., of the kidneys or liver.

Of the large variety of known mycotoxins, the major *Fusarium* spp. mycotoxins are, apart from AFs, the most common and harmful mycotoxins. *Fusarium* spp. produce three of the most important classes of mycotoxins with respect to human health; zearalenone (ZEA), trichothecenes (deoxynivalenol (DON), T-2 and HT-2 toxins) and fusarium, nivalenol (NIV); but *Fusarium* spp. genera also produce emerging mycotoxins, such as beauvericin (BEA), enniatins (ENNs) and moniliformin (MON), which are more recently discovered and less studied [5] (Table 1).

In recent years, interest in mycotoxins as neurotoxins has increased, becoming the cause of numerous neurological diseases and illnesses, which is confirmed by numerous scientific and epidemiological studies. Of particular importance is the widespread contamination of food and feed with *Fusarium* mycotoxins, which contribute to disorders of the nervous system. The publication aims to indicate the existing danger of neurotoxicity of these mycotoxins. Recent in vitro and in vivo studies prove that *Fusarium* mycotoxins cause neurotoxicity and are responsible for the development of neurodegenerative diseases. Many researchers have indicated an important mechanism of mycotoxin neurotoxicity, which is oxidative stress and lipid peroxidation (LPO) leading to changes in the transmitter system by destroying membrane receptors. Additionally, the harmful effect of mycotoxins on DNA and RNA important in the development of neurotoxicity is the possibility of causing changes in the permeability of amino acids along the entire blood–brain barrier (BBB) in the mother and in the fetus, leading to its death as a result of fetotoxicity mainly in the central nervous system (CNS) [6,7]. Mycotoxins’ neurotoxicity and cytotoxicity have been demonstrated by the impairment of gene expression, cell proliferation and the induction of oxidative stress. The multilayered mitochondrial network of antioxidant systems, consisting of many enzymes—glutathione transferase (GST), glutathione peroxidase (GPx), catalase (CAT), superoxide dismutase (SOD) and glutathione reductase (GR)— protects cellular systems [8,9,10,11,12,13].

However, mycotoxins produced by *Fusarium* spp. are widely studied as one of the factors triggering cell death. Mycotoxins cross the BBB, causing neuronal damage, brain inflammation and neurochemical imbalances. Studies conducted on animals and humans have confirmed the correlation between high concentrations of mycotoxins in the blood and neurological damage affecting cognitive skills, anxiety and other states [14,15,16,17,18,19].

*Fusarium* mycotoxins have also been shown to influence the enteric nervous system (ENS) by affecting regulatory processes in the gastrointestinal tract, but they also cause adaptive and protective responses [20]. It has been shown that changes in the ENS are the first symptoms of toxicity at low doses of DON [21]. The influence of low doses of T2 toxin on various intestinal functions, such as motility, secretion, the conduction of sensory stimuli and the regulation of blood flow in the intestinal wall has been described [22,23,24]. Low doses of ZEA affect the neurochemical coding of nerve fibers in the mucosal and muscular layers of the intestine [24,25,26]. The effect of fumonidins on the ENS depends on the gastrointestinal segment and the type of intestinal plexus [27,28].

Epidemiological data have shown that concentration of the T-2 toxin in the serum of 182 residents with a history of exposure to mycotoxins was significantly increased, neurodevelopment was hindered and exercise balance, visual function, reaction time, cognitive abilities and emotional management skills were impaired. Additionally, a decrease in frontal cortex activity was detected through quantitative EEG [29,30]. With individual neuropsychological data and symptom observations, it was also suspected that these factors contribute to autism spectrum disorders, consistent with recent epidemiological studies linking mycotoxins to autism spectrum disorders [31,32]. Neurophysiological studies in children including electroencephalogram (EEG), brainstem evoked potential (BAEP), visual evoked potential (VEP) and somatosensory evoked potential (SSEP) showed that evoked potentials from the brainstem, vision and somatosensory pathways were abnormal after prolonged exposure to mycotoxins [33,34]. Cognitive impairment in 277 children in Poland was evidenced by a 6-year mycotoxin exposure test, indicating that the duration of exposure was negatively correlated with the participants’ IQ. Similarly, the concentration of OTA in the urine and serum of 52 patients with autism was significantly higher than in the control group. Furthermore, the inclusion of microRNA-132 driven by OTA in vitro was verified, which was associated with depression-related genes PTEN and MeCP2. Conversely, the method of adsorbing or removing OTA could alleviate autism symptoms [27,35,36]. Based on the characteristics of these common mycotoxins regarding their ease of crossing the BBB and their potential threat to the CNS, studying the interconnections between their neurotoxic mechanisms and nerve-related diseases becomes increasingly urgent for us.

The mechanism of neurodegenerative diseases has been shown to be associated with the disturbance of both sphingolipid and glycerophospholipid metabolism and cysteine and methionine metabolism [37,38]. Epidemiological data have shown that natural antioxidants are used in mitigating the neurotoxicity of mycotoxins. Additionally, low molecular weight antioxidants (ubiquinol and α-tocopherol) and those found in food (lutein, zeaxanthin and polyphenols) have a similar protection function regarding the neurotoxicity of mycotoxins [39].

The neurotoxicity of *Fusarium* mycotoxins is an interesting research target, taking into account the relationship with neurodegenerative diseases as well as focused research on the development of neuroprotective drugs or the development of preventive measures. The article aims to present the threats posed by common *Fusarium* mycotoxins in food as factors causing CNS disorders and methods of preventing and minimizing the effects of mycotoxin neurotoxicity.

## 2. Results

According to a study conducted by the Food and Agriculture Organization of the United Nations, the *Fusarium* mycotoxins are the most important fungal toxins in economic terms [40,41]. Zearalenone, fumonisins and trichothecenes (T-2 toxin, HT-2 toxin, DON, NIV, etc.) are the main representatives and most frequently detected *Fusarium* mycotoxins in cereal grains and food/feed. They cause huge economic losses and pose a threat to human and animal health worldwide.

### 2.1. Zearalenone (ZEA), α-Zearalenone (α-ZEL) and β-Zearalenone (β-ZEL)

The main mycotoxin causing fertility disorders in humans and animals is ZEA and its main metabolites: α-ZEL and β-ZEL. ZEA is a nonsteroidal estrogen-like mycotoxin that exhibits the ability to completely bind to estrogen receptors (ER) ER-α and ER-β [42,43]. ZEA can undergo the reduction of the keto group on C-8 into diastereoisomeric ZEA (α and β). α-ZEL, which is about three times more estrogenic than ZEA, is the main metabolite formed in pigs and humans. However, in addition to being key hormonal regulators of reproductive function, estrogenic compounds play a widespread role in the brain as factors influencing the activity of several brain areas that are not directly related to reproduction. Therefore, this review will begin by presenting data indicating the action of ZEA and its metabolites on some neuronal functions.

**Figure 1:**
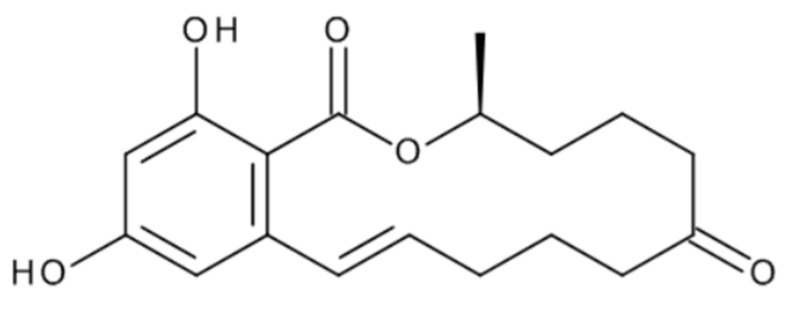
Chemical structure of ZEA.

ZEA, chemically described as 6-(10-Hydroxy-6-oxo-trans-1-undecenyl)-beta-resorcylic acid-N-lactone (Figure 1), is a secondary metabolite of fungi of the genus *Fusarium* spp. The largest amounts are produced by fungi of the species *F. graminearium*, *F. crookwellense* and *F. culmorum*. ZEA is mainly detected in corn and other cereals and their products. The daily intake for ZEA is 0.25 µg/kg body weight (b.w.) and was established by the EFSA Panel on Contaminants in the Food Chain in 2011. The established tolerance is based on estrogenicity in pigs. No new studies were identified that could change the daily intake [10]. ZEA, depending on scientific reports, was frequently detected in various cereals such as rice, wheat, barley, hay, corn, sorghum, rye, corn silage, sesame seeds, flour, soybeans and corn oil [15,16,29].

ZEA affects neurons in the central nervous system by crossing the BBB [39,40]. Several studies have proven that exposure to this mycotoxin leads to the abnormal synthesis of enzymes and neuronal factors in brain neurons. In addition, it increases oxidative stress reactions, induces apoptosis of neuronal cells and affects the development of the nervous system. ZEA affects the functions of glial cells and can also cause behavioral aberrations [39,40,41,42,43,44].

In the study by Venkatarman et al., the role of ZEA as a mediator between oxidative stress and the human neuroblastoma cell line SH-SY5Y was analyzed [40]. The discussed mycotoxin increased the level of free malondialdehyde (MDA) and induced the generation of free oxygen radicals (ROS). The loss of mitochondrial membrane potential (MMP) was also associated with the action of ZEA. Moreover, ZEA was shown to be responsible for the increase in DNA damage in a dose-dependent manner. In the plasmid disruption assay, no DNA damage was observed at specific time points of 6, 12 and 24 h. Apoptotic nuclei were demonstrated in DAPI staining after 12 and 24 h. SH-SY5Y cells treated with the mycotoxin ZEA showed a clear inhibitory effect on the expression of neuronal genes [40].

From a practical point of view, since ZEA and DON occur together in contaminated cereals and both have neurotoxic potential, additional reports on the toxic effects of *Fusarium* toxins on the nervous system concern ZEA and DON and are based on the study of mouse brain mass, antioxidant and apoptosis indices. In both analyzed groups, after 3 and 5 days, higher levels of total nitric oxide synthase, nitric oxide, hydroxyl radical scavenging and MDA were noted. At the same time, the levels of brain proteins, glutathione (GSH), SOD, GPx activity and the percentage of apoptotic cells were reduced. By day 12, most of the described indices had returned to normal levels [45]. The mechanism of the neurotoxic action of ZEA is closely related to the increase in oxidative stress in cells. Concerning its metabolites (α-ZEL, β-ZEL), which also exhibit neurotoxic effects, the very similar mechanism of toxic action on nerve cells has been demonstrated (Table 2).

Agahi et al. in their study evaluated the effect of mycotoxins α-ZEL and β-ZEL, which are metabolites of ZEA and BEA, in combination and individually on the enzymatic antioxidant activity of GST, GPx, CAT and SOD. The authors analyzed the enzymatic protection system in an undifferentiated human neuroblastoma cell line—SH-SY5Y. In the undifferentiated form, these cells have catecholaminergic characteristics and are morphologically recognized by similar neuroblasts [46]. In various studies determining the pathogenesis of neurotoxicity, neuroblastoma (SH-SY5Y) is used as a cell model [47,48,49]. The discussed mycotoxins, both in combination and individually, increase the activity of GPx in the undifferentiated SH-SY5Y cell line after 24 h of exposure. The authors suggest that the production of ROS began under the same conditions in their previous study [50], which may be related to the reduced effects of mycotoxins α-ZEL, β-ZEL and BEA. On the other hand, the decrease in the activity of GPx and CAT enzymes was related to the high activity of SOD detected after 48 h. The researchers indicate that the CAT enzyme in SH-SY5Y cells after combined and single exposure showed the highest activity only for two tested mycotoxins—α-ZEL and β-ZEL. The activity of GST increased when mycotoxins were combined, which proves that GST activity tries to alleviate the damage associated with oxidative stress. In summary, the studies conducted by Agahi et al. show that the antioxidant enzyme system in SH-SY5Y neuroblastoma cells plays a strong protective role against damage caused by α-ZEL, β-ZEL and BEA mycotoxins both when combining the discussed mycotoxins and in a single study [51].

In their second work, Agahi et al. studied the effect of α-ZEL, β-ZEL and BEA on the production of ROS in the human neuroblastoma cell line SH-SY5Y. The aim of the study was to analyze the progression of the cell cycle and the cell death pathway by flow cytometry for 24 h and 48 h individually and in combination in binary and tertiary mixtures. In the case of β-ZEL, significant changes in the cell cycle were observed at the two analyzed times—arrest or delay in the G2/M and S phases and activation of cell proliferation in the G0/G1 phase. This effect was not observed after exposure to α-ZEL. Triple mixtures caused a 4-fold increase in cell proliferation in the subG0 phase compared to the control. β-ZEL alone at the highest concentration and in combination therapy caused a significant increase in the population of early apoptotic cells in the studied early apoptosis and cell apoptosis/necrosis pathways. However, in the case of α-ZEL, apoptotic cells were shifted to late apoptotic cells after 48 h of exposure [52]. Similar conclusions were drawn by others study macrophages and showed a significant increase in the number of apoptosis cells after exposure to β-ZEL compared to α-ZEL [52], and by Zhu et al., who proved that β-ZEL causes cell death via the apoptotic pathway in porcine granulosa cells [53].

**Figure 2:**
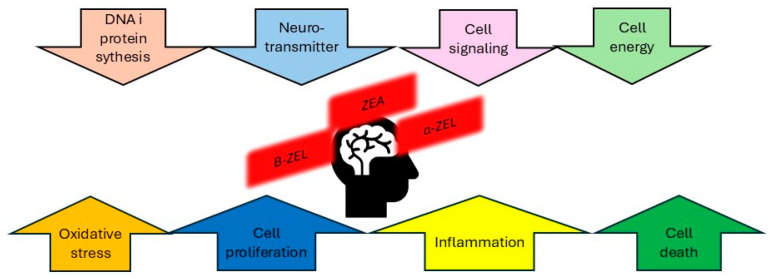
The general mechanisms affected by the mycotoxins ZEA, α-ZEL and β-ZEL are shown based on published data generated in neuronal cultures or collected in vivo by examination of the brain.

In conclusion, mycotoxins α-ZEL, β-ZEL and BEA affect the molecular mechanisms of cell death in the nervous system (Figure 2). More extensive studies are needed to explain the new approach to preventing neurotoxicity.

At this point, it is worth presenting the results of the study by Agahi et al., which analyzed the neuroprotective effect of *Allium sativum* L. (Voghiera garlic) extract, a local garlic ecotype from Italy, on undifferentiated SH-SY5Y neuronal cells against α-ZEL, β-ZEL and BEA. The results showed a significant improvement in cell viability in the strategy of pre- and co-treatment with Voghiera garlic extract (VGE), especially in the case of co-treatment with β-ZEL and then with α-ZEL and BEA. Separate treatment with VGE with each tested mycotoxin decreased significantly. The results of the study do not explain the protective mechanisms of garlic in the case of toxicity of the studied mycotoxins. An attempt was made to interpret the protective effect of garlic in relation to the toxicity of other mycotoxins, such as T-2 or OTA. The protective effect of garlic in T-2 and OTA toxicity consisted in reducing oxidative stress, GSH and GPx activity. On this basis, it was concluded that garlic extract is effective in reducing the neurotoxicity of α-ZEL, β-ZEL and BEA mycotoxins [54].

### 2.2. Trichothecenes T-2 and HT-2

**Figure 3:**
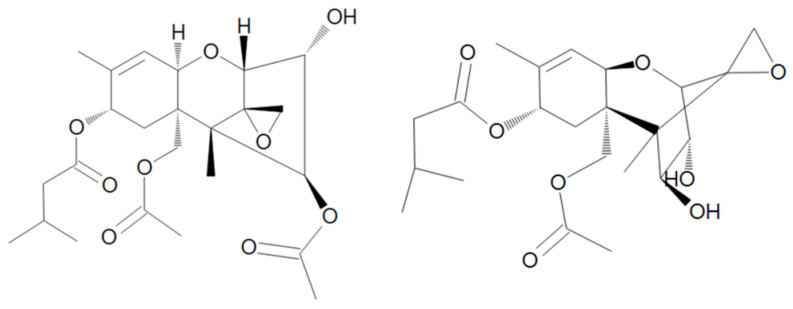
Chemical structure of T-2 and HT-2.

**Figure 4:**
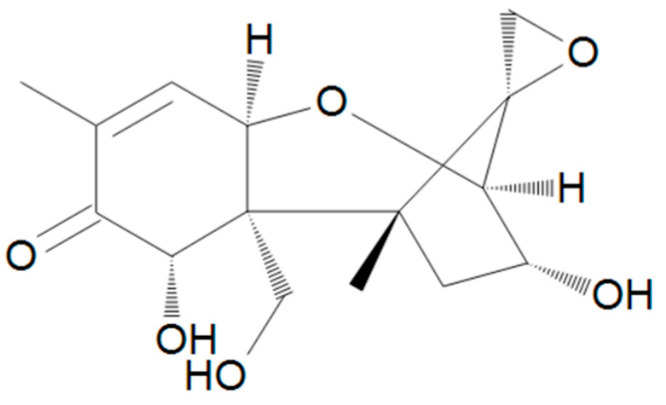
Chemical structure of DON.

Trichothecenes are sesquiterpenoid compounds with an epoxy group at C12-13 that is considered essential for toxicity. Although more than 100 trichothecene mycotoxins have been identified, type A trichothecenes include some of the most toxic trichothecenes, such as T-2 toxin and its deacetylated metabolite HT-2 toxin (Figure 3). Type B trichothecenes are common field contaminants of grains and include DON (or vomitoxin) (Figure 4).

In recent years, the European Food Safety Authority (EFSA) has defined the tolerable daily intake of the sum of T-2 trichothecene mycotoxins produced by *Fusarium* spp. and its metabolite HT-2 at the level of 100 ng/kg [55]. These toxins require moist and cool conditions for their development, which is why they are mainly found in oats, barley and wheat [56]. Exposure to T-2 toxin is associated with a wide range of neurological symptoms—ataxia, anorexia and muscle weakness [57,58]. Many scientific reports indicate the mechanisms of T-2 toxin neurotoxicity, i.e., oxidative stress, ROS, mitochondrial dysfunction, increased apoptosis [18].

In one study, rats orally administered T-2 showed reduced performance in the passive avoidance test and reduced motor activity [59]. Guo et al. had noticed changes in the levels of monoamines in specific areas of the rat brain [58]. Another report performed in vivo in pregnant rats showed the induction of apoptosis in the fetal brain on day 13 of pregnancy [59]. Moreover, after subcutaneous administration of T-2 in adult mice, a change in the permeability of the BBB was noted [11].

Weidner et al. assessed the apoptotic effect of T-2 toxin on human astrocytes by caspase-3 activation, annexin V staining and lactate dehydrogenase (LDH) release. Human astrocytes in primary culture (NHA) were used in the study. The cytotoxicity, apoptosis, necrosis and metabolism of T-2 toxin in NHA were examined and compared with the uptake in an established human cell line (HT-29) [60]. Cellular uptake was also taken into account. Six hours after exposure to mycotoxin, a significant increase in caspase-3 activity was observed. Also, the uptake study on human astrocyte cells (NHA) and a human cell line (HT-29) showed high accumulation in both cell types and rapid cellular uptake, which causes a 15–30-fold increase in concentration in the intracellular compartment. Human astrocytes show high sensitivity to T-2 toxin. This induces strong cytotoxic responses after just a few hours of incubation at low toxin concentrations. At the same time, it is worth noting that in the cited study, the formation of the main metabolite of T-2 toxin—HT-2 toxin—was detected in both types of cells studied, i.e., HT-29 and NHA. It seems that it occurs inside the cells. When assessing the risk of neurodegeneration, both compounds should be considered, because the observed apoptotic effects are probably caused by the combination of both toxins [60].

Guo et al. analyzed the effect of T-2 toxin on the central nervous system. Female rats were given a dose of 2 mg/kg b.w. of T-2 toxin and sacrificed after 1, 3 and 7 days. Histopathological analysis of the brains and pituitary glands was performed. Changes in the brain were visible after 3 days of exposure to T-2, and in the pituitary gland after 7 days. Autophagy in the brain and apoptosis in the pituitary gland suggest that T-2 toxin may induce different acute reactions in different tissues by directly crossing the BBB [58].

### 2.3. Deoxynivalenol (DON)

Another type B trichothecene mycotoxin is DON, which is mainly produced by *Fusarium* spp. and is commonly found worldwide [61,62]. The daily tolerance dose of this mycotoxin is 1 μg/kg body weight per day, which was established for DON based on reduced body weight gain in mice [63]. DON is mainly found in maize, wheat and rice [64].

The changes induced by this mycotoxin in the nervous system include impaired neuronal activity and the abnormal synthesis of neuromodulators and/or neurotransmitters [65,66]. DON is also responsible for the induction of apoptosis and disorders related to calcium homeostasis in neurons and LPO [66]. The brain contains high levels of catecholamines, unsaturated fatty acids, and is, therefore, susceptible to oxidative damage. The most sensitive species include pigs, then cats, rodents, dogs and ruminants [67,68,69].

DON affects the secretion of neurotransmitters in the brain in vivo, such as 5-hydroxytryptamine (5-HT, serotonin), which causes vomiting in pigs [68]. A dozen or so years ago, it was also proven that DON causes an increase in norepinephrine levels, which results in a decrease in dopamine levels in piglets [69]. In turn, Adelsberger et al. indicate the disruption of Ca^2+^ homeostasis, which causes abnormal or reduced generation of functional neuronal circuits in the maturing hippocampus [70]. Wang et al. supported this thesis by indicating the association of DON with a decrease in mRNA and protein expression of calmodulin, which is a calcium-binding signal transducer [61]. Furthermore, DON contributes to oxidative stress in HEK-293 and DF-1 cells [71,72]. Wang et al. studied the effect of DON on the nervous system. First, they indicated the correlation of DON with the hippocampus. During treatment with the discussed mycotoxin, the researchers observed chromatin aggregation and condensation, the lack of nuclei and severe cytoplasmic edema and the lack of nuclear and subcellular organelles and mitochondrial edema with increased DON concentration [73].

Depending on the concentration, DON also inhibited the activity of GPx and SOD in the cerebellum, cerebral cortex and hippocampus. The described study confirmed the reports of other authors on the correlation of DON with 5-HT serotonin. The concentration of this neurotransmitter increased in all tested tissues during mycotoxin treatment. The level of 5-HT serotonin significantly increased in the hippocampus and cerebellum compared to the control sample [73]. Other reports have reported patterns of DON cytotoxicity based on the time of ROS generation, DON dose, LPO, loss of MMP, intracellular GSH release and DNA damage in SH-SY5Y cells. They also indicate a reduction in the number of genes for neurotrophic factors such as TH, BDNF and AADC, which makes DON-induced cell death associated with the impaired regulation of neurotransmitter release and reduced neuronal function [74].

The number of studies focusing on the mitigation of DON-induced cytotoxicity is limited. Dietary antioxidants (e.g., quercetin) have been shown to have oxidant-scavenging effects, which reduce inflammation and apoptosis [75]. Quercetin (QUE) is a bioflavonoid and is commonly found in some fruits and vegetables, including apples, figs, grapes, citrus fruits, buckwheat and black and green tea [76]. Quercetin has been shown to have neuroprotective, cardioprotective, vasoprotective and anticancer effects [75,76]. There are no reports on the use of QUE as a cytoprotective agent against DON-mediated oxidative imbalances.

### 2.4. Beauvericin (BEA), Enniatin A (ENN A) and Enniatin B (ENN B)

**Figure 5:**
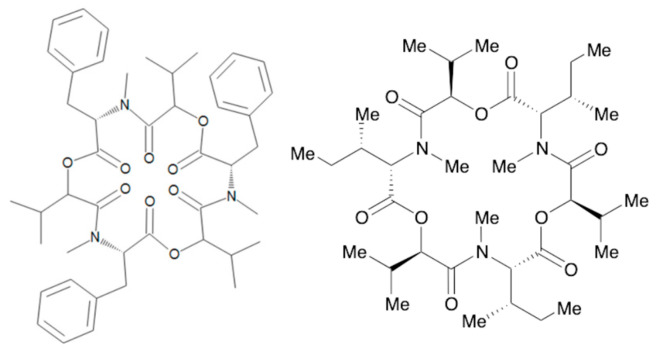
Chemical structure of BEA and ENN.

The group of mycotoxins classified as “emerging mycotoxins” includes secondary metabolites of *Fusarium* spp.—BEA and enniatin A and B (ENN) [77,78,79]. Chemically, BEA and ENN are cyclic lactone trimers of amide of N-methyl l-phenylalanine and d-α-hydroxyisovaleric acid (Figure 5). This mycotoxin probably acts as an ionophore capable of making complexes with divalent cations. Data from the EFSA report the presence of significant amounts of BEA and ENN—or more precisely, four types, A, A1, B and B1—in cereal grains. However, the regulations do not regulate the content of BEA and ENN in food. According to the EFSA, human health should not be endangered by the acute exposure to ENN [78]. The presence of ENN B and BEA has also been reported in cow’s milk [79].

BEA is attributed with the ability to cause oxidative stress, produce ROS and cause cytotoxicity, as well as the ability to peroxidize lipids [54].

ENN, and especially, enniatin B and B1, is among the most common mycotoxins, indicating the significant contamination of feed with *Fusarium* spp. Studies have shown the presence of ENN B and B1 in urine, blood and even breast milk, which indicates that as humans, we are exposed to this mycotoxin [79,80,81]. ENNs are composed of alternating d-2-hydroxyisovaleric acids and N-methyl-1-amino acids (Figure 5). Nearly 29 structural analogues of these cyclic hexadepsipeptides have been described [82,83,84].

Studies have shown that compounds with a similar structural structure to ENN are able to easily enter the mammalian body. In an in vitro study simulating the duodenum and large intestine, it was determined that ENN A, ENN B and B1 were characterized by the highest bioavailability [85]. Importantly, it seems that the skin may also not protect against the penetration of mycotoxins into the body. In the study by Meca et al., the ability of ENN and BEA to penetrate undamaged and damaged skin was assessed using Franz diffusion cells in vitro. It was shown that the skin does not constitute a barrier against the penetration of these substances, determining that ENN B is characterized by the highest permeability, and BEA by the lowest [85].

In light of the conducted studies, the ability of these compounds to penetrate the BBB and their potential neurotoxicity is intriguing. In the study by Krug et al., the effect of ENN B and ENN B1 on BBB cells was assessed using three types of cells: HBMEC (human brain microvascular endothelial cells), PBCEC (porcine brain capillary endothelial cells) and CCF-STTG1 (human astrocytoma cells). It was determined that the effect of the tested substances on the viability of brain HBMEC was less pronounced than in CCF-STTG1; however, both mycotoxins induced high cytotoxicity in CCF-STTG1. The tested ENNs, especially, enniatin B, led to the induction of apoptosis rather than necrosis of CCF-STTG1 cells. This study demonstrated the high permeability of ENN B and ENN B1 from blood to the brain via passive transport. It was determined that the efflux of ENN from the basolateral to the apical compartment during active transport is weak [84]. Also, a previous in vivo study in mice, determining the penetration of BEA and ENN across the BBB, indicated a rapid and large influx of these mycotoxins into the brain of the animals tested [86].

In addition, genotoxic effects were observed in mice after repeated oral exposure to BEA and ENN B. The EFSA predicts that “emerging mycotoxins” may become a public health problem [87].

In another study, 48 h exposure of a human neuroblastoma cell line (SH-SY5Y) to BEA caused a significant decrease (by 43%) in cell viability compared to unexposed cells [54].

In the study by Pérez-Fuentes et al., the effect of different mycotoxins (DON, ZEA, fumonisin B1, B2 (FB1 and FB2), BEA and ENN) on the viability and mitochondrial function of human neuroblastoma cells (SH-SY5Y) was analyzed. ENN, BEA and DON showed the greatest harmfulness to the tested cells (IC_50_ of 0.35–2.4 µM). It was observed that “non-regulated mycotoxins”, by affecting the MMP, can affect cell apoptosis, and antagonistic effects of mixtures of “non-regulated” and “regulated” mycotoxins were also noted. Scientists claim that “emerging mycotoxins” can be more neurotoxic and also impair mitochondrial function to a greater extent than regular mycotoxins [88]. Scientists also analyzed extracts of cow feed, identifying “regulated” and “emerging” mycotoxins. Cytotoxicity and reduced viability were observed in neuroblastoma cells treated with the extract, despite the fact that the “regulated mycotoxins” were present in lower concentrations than the EU recommends for animal feed (ENN and BEA were also detected in small amounts). Scientists suspect that this harmful effect may be caused by the presence of other mycotoxins, i.e., ENN A1, ENN B1 and FB2—enhancing the effect of those selected for the study. Two milk samples were also examined—both samples were similarly contaminated with BEA and ENN in low concentrations, but the second sample contained ENN B1. The first sample did not affect SH-SY5Y cells, while the second reduced their viability, which indicates that ENN B1 may have neurotoxic properties and enhance the effect of other mycotoxins. Scientists suspect that the harmful effect of “emerging mycotoxins” may be related to their lipophilicity [88].

### 2.5. Mycophenolic Acid (MPA)

**Figure 6:**
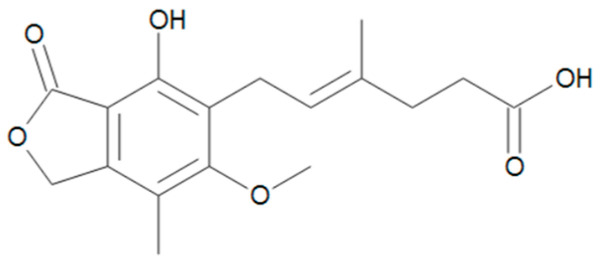
Chemical structure of MPA.

MPA is a mycotoxin produced by the fungus *P. roqueforti* [89,90]. The mycotoxin is a member of the class of 2-benzofurans that is 2-benzofuran-1(3H), one which is substituted at positions 4, 5, 6 and 7 by methyl, methoxy, (2E)-5-carboxy-3-methylpent-2-en-1-yl and hydroxy groups, respectively (Figure 6). Its presence was demonstrated, among others, in maize silage in a study conducted in Norwey by Uhlig et al. MPA was present in 74 of 233 samples analyzed [91]. Although the mechanism and strength of the toxic effect of MPA are still not fully understood, scientists suggest caution in feeding farm animals with feed contaminated with *P. roqueforti*, as it is very likely to have a negative effect on some body systems, including the nervous system, due to the deposition of its toxins in tissues. Studies on cultured neuro-2a cells derived from a spontaneous neuroblastoma tumor exposed to *P. roqueforti* and, consequently, MPA demonstrated impaired mitochondrial function, the depletion of ATP reserves and the production of ROS [89,90].

### 2.6. Moniliformin (MON)

**Figure 7:**
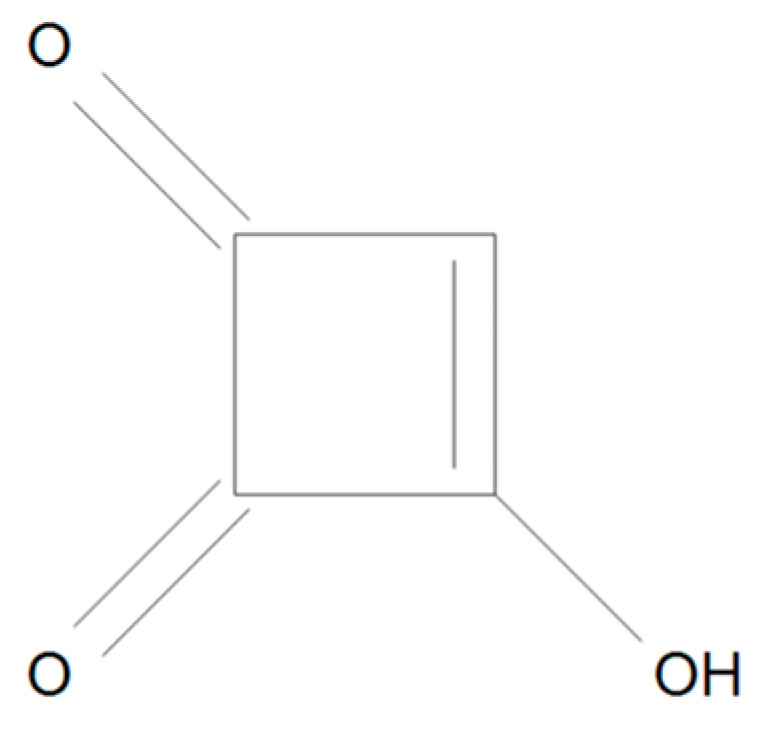
Chemical structure of MON.

MON is a secondary product of fungi of the *Fusarium* genus. MON, a mycotoxin produced by *Fusarium* spp., is a small polar molecule possessing a cyclobutanedione structure. It occurs as a sodium or potassium salt of 1-hydroxycyclobut-1-ene-3,4-dione (Figure 7). Its highest concentrations are found in contaminated cereals [92,93]. A Norwegian study showed that MON was present in 25, 32 and 76% of barley, oat and wheat samples, respectively. The maximum concentrations of this mycotoxin in the cereals tested were 380, 210 and 950 μg/ kg [91]. Behrens et al. conducted an in vitro study on the permeability of mycotoxins, including MON, across the BBB on primary porcine brain capillary endothelial cells (PBCEC) [92]. In addition to MON, DON and its derivative 3-acetyldeoxynivalenol (3-AcDON) were also studied. MON, due to its small molecular size, low mass and high polarity, is a compound difficult to analyze quantitatively, which is why there are few studies that could explain its mechanism of action and its neurotoxicity [92,94,95]. The above study showed that the incubation of 10 μM MON did not affect the cell viability of PBCEC for up to 48 h. MON also has no significant effect on the integrity of cell membranes and increased permeability of the PBCEC monolayer. It was also found that MON is characterized by the same rate of BBB penetration as DON and morphine, which has a strong effect on the CNS, but both MON and DON penetrate the BBB only to a small extent. In contrast, 3-AcDON is characterized by high BBB permeability and may release the more toxic DON as a result of hydrolysis [94,95,96]. It should be noted that the study indicates that MON and 3-AcDON have weak or almost no adverse effects on the BBB in vitro [92].

Other scientists, led by Jonsson, conducted studies on the oral toxicity of MON in Sprague-Dawley rats at a dose of 25 mg/kg body weight. They found that 38% of the administered substance was detectable in urine 6 h after MON administration, while at the end of the study, 42% was observed in the urine and less than 1% in the feces. These results indicate high bioavailability and suggest that MON not excreted from the body may accumulate and undergo further metabolism in rat tissues [95]. In turn, in another study by Jonsson et al. conducted on the same rat model, they analyzed the lowest toxic concentration of MON given by LOAEL of 3 mg/kg body weight. It was established that 20.2–31.5% of the administered dose of MON is excreted in urine, while 2% of the administered dose was detected in feces. However, the scientists did not find any signs of MON accumulation [96].

It should be assumed that MON, due to its high bioavailability and some permeability to the BBB demonstrated in in vivo studies [92,96], may affect nervous tissues. However, previous in vivo and in vitro studies on MON show discrepancies regarding its toxic effects, which requires further and broader experiments and interpretation of the results [77,92,96]. It can be assumed that, because of the structural similarity to pyruvate, MON affects energy metabolism via the inhibition of mitochondrial pyruvate and a-ketoglutarate oxidation during the Krebs cycle. MON is also thought to inhibit other metabolic pathways involving pyruvate. It has been reported that MON inhibits the free radical scavenging enzymes GPx.

It is important to note that the permeability of mycotoxins, which, as single compounds, cannot penetrate the BBB, may be influenced by the supporting action of other substances including trichothecenes, which include DON and, by impairing the continuity of the barrier, enable them to reach the brain [92].

### 2.7. Sterigmatocystin (STG) and Cyclopiazonic Acid (CPZ)

At the end of the article, we would like to present two interesting mycotoxins, namely, STG and CPZ, which do not belong to *Fusarium*. Initial studies indicate their high neurotoxic activity.

**Figure 8:**
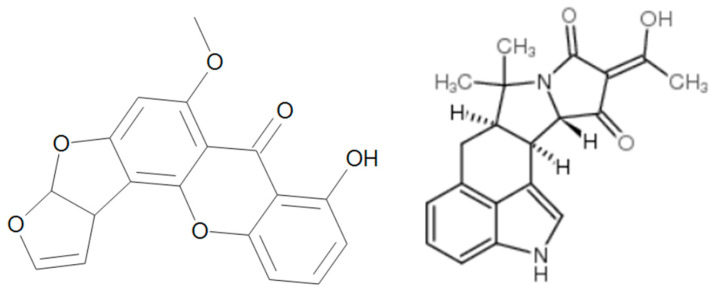
Chemical structure of STG and CPZ.

STG consists of a xanthone nucleus attached to a bisfuran structure (Figure 8). The acute toxicity of STG is low, and the main concern is that it is highly neuro-carcinogenic as AF.

De Sa et al., in their study, analyzed exposure to mycotoxins commonly found in consumed cereal products, including STG and CPZ, as well as ENNB, aflatoxin B1 (AFB1), citrinin (CIT) and OTA [97]. The STG and CPZ that are of interest to the authors are produced mainly by fungi of the *Aspergillus* spp. Cytotoxicity in human neuroblastoma cells (SH-SY5Y) was assessed. These mycotoxins are widespread in food products, especially cereals and products intended for infants and children—cereal flakes, cookies and rice—which all the more raises the need to investigate their effects on the body. Scientists also examined the mutual effects of mycotoxins, checking their synergistic or antagonistic effects. The highest single toxicity was characteristic of ENNB (IC_50_—inhibitory concentration = 3.72 μM), followed by OTA (9.10 μM) and STG (9.99 μM). On the other hand, the highest toxicity of mycotoxins acting together was demonstrated by the combination of OTA + STG (IC_50_ = 3.77 μM). The combination of CPZ + CIT had the least harmful effect. Most of the analyzed combinations showed synergistic or additive effects, but the combinations of ENNB + STG, ENNB + AFB1 and CPZ + CIT showed antagonistic interactions [98].

Zingales et al. point to the harmful effects of STG on the cells of the nervous system, indicating its effect promoting oxidative stress [98,99]. STG is also responsible for LPO and inhibition of mitosis and the cell cycle [100].

Another dangerous compound belonging to the “emerging mycotoxins” is CPZ [97]. Chemical CPT is an indole tretraminic acid. It has been classified by EFSA as a neurotoxic substance, causing neurotoxic and degenerative effects during chronic exposure, as well as symptoms such as ataxia or spastic paralysis, in some cases leading to death [100,101,102].

The basis for the negative effect of CPZ on cells is the intensified and selective inhibition of calcium ion flow, mainly by inhibiting the activity of calcium ATPase [97]. Studies by de Sa et al. indicate the need for further analyses of mycotoxin interactions and adverse effects on the human body due to their wide distribution and mutual occurrence in contaminated food products [97].

### 2.8. Mechanism of Fusarium Mycotoxins Neurotoxicity

Exposure to *Fusarium* mycotoxins can disrupt the biochemical and molecular functions of the CNS (neurotoxicity). Food-origin mycotoxins, including T-2 toxin, DON, ZEA, OTA and FB1, have been implicated in neuronal and brain damage [1]. These toxins can infect astrocytes and microglia, potentially contributing to neurodegenerative diseases like Alzheimer’s and Parkinson’s [103]. The easy passage of these mycotoxins through the BBB underscores the need to explore their neurotoxic mechanisms and connections to nerve-associated diseases [1]. The found that the inhibition of protein synthesis leads to neuronal and neurochemical disorders and many brain dysfunctions. An important cause of mycotoxin neurotoxicity is oxidative stress, which affects neuronal membranes. Mycotoxins disrupt the release and reuptake of neurotransmitters such as dopamine, GABA and serotonin, causing brain dysfunction. Among the neurotoxic effects of mycotoxins, DNA damage should also be mentioned, which leads to genetic mutations that can contribute to neurological diseases. According to the literature data, oxidative stress induced by mycotoxins is a major factor in nerve cell damage [103]. This is confirmed by the studied indicators of oxidative stress in nerve cells (lipoperoxidation, MDA or GSH levels) after exposure to mycotoxins. Therefore, substances that can increase antioxidant defense and minimize damage will be crucial for protection [104,105]. Mycotoxins can trigger an inflammatory response in the brain, including the release of proinflammatory cytokines and other mediators, which have been associated with various neurological conditions. In addition, mycotoxin exposure can lead to an imbalance of neurotransmitters in the brain, affecting communication between neurons and leading to adverse neurological symptoms. Another important biochemical cause of mycotoxin-induced CNS toxicity is mitochondrial dysfunction. Mycotoxins reduce energy availability for neurons and impair brain function. The effects of mycotoxin exposure on the CNS may vary depending on the type of mycotoxin, the dose, the duration of exposure and individual susceptibility. Furthermore, it has been shown that the exposure of animals to mycotoxins can cause behavioral disorders, in particular, cognitive deficits and anxiety-like behaviors [106,107,108]. The causes of such symptoms are oxidative and inflammatory damage, leading to mitochondrial dysfunction and neurochemical injury. Key enzymes such as AChE and monoamine oxidase isoforms are damaged, indicating possible dysfunctions in cholinergic and monoaminergic signaling. Furthermore, cell cultures of neurons exposed to mycotoxins have shown neurotoxicity, impairing excitatory and inhibitory neurotransmission [109]. The effective scavenging of lipid superoxide radicals prevents free radical chain reactions of LPO [29]. Mitochondria also convert about 5% of oxygen in cells into ROS. They cannot be fully neutralized by defense systems [33]. All these aspects are related to the development of neurodegenerative diseases, enhancing or mediating neuronal dysfunction during neurodegeneration [29,33]. Therefore, mycotoxin toxicity to the central nervous system and its contribution to the development of neurodegenerative diseases appears to involve multiple mechanisms of action, complicating the treatment of poisoning.

### 2.9. Methods to Reduce Mycotoxin Neurotoxicity

Studies conducted to minimize toxicity, especially neurotoxicity, caused by mycotoxin exposure have shown that treatment based on natural compounds was effective [104,105,106,107,108,109,110,111,112,113,114,115]. Studies have mainly focused on the use of isolated phytochemicals classified as polyphenols. These substances possess antioxidant and anti-inflammatory properties, which have been widely demonstrated in the scientific literature [104,105,106,107,108,109,110,111,112,113,114,115]. In addition to the use of isolated phytochemicals, some studies have successfully used honey [110], oils [111,112,113,114] and plant extracts [115,116,117,118]. These substances were evaluated for their chemical composition and found to contain polyphenols, which indicated their potential in the treatment of mycotoxin poisoning. Honey is a source of natural antioxidants [110]. Honey is mainly composed of carbohydrates, which constitute about 95% of its dry weight. In addition to carbohydrates, it also contains various other substances such as organic acids, proteins, amino acids, minerals, polyphenols, vitamins and aroma compounds. In addition, honey is rich in flavonoid components. Therefore, the potential therapeutic applications of honey in mycotoxin poisoning were investigated. The study was conducted on Sprague-Dawley rats, which were given aflatoxin (25 ug/rat/day) and honey 1 mL/kg b.w. in the diet for over 90 days. It was found that adding honey to the diet reduced LPO and levels of enzymes related to liver damage while increasing enzymatic and non-enzymatic antioxidants in rats that received both honey and AFs. The protective effect of honey on the liver and kidneys was further confirmed by histopathological examination of the liver and kidneys of the group of animals receiving honey and AFs, in contrast to the degenerative changes in these organs in rats that received only AFs. In addition, honey supplementation improved antioxidant defense systems and LPO in other tissues of animals that received both honey and AFs. However, further studies are needed to confirm the antioxidant properties of honey and to clarify its potential effect in the treatment of *Fusarium* mycotoxin poisoning [110].

It has been shown that dietary supplementation with oils can reduce the degree of tissue damage caused by mycotoxin exposure. Eraslan et al. aimed to determine whether pumpkin seed oil (1375 mg/kg.b.w./d, orally, for 21 days) could improve mycotoxin-induced LPO and ROS [111]. Pumpkin seed oil contains compounds such as vitamin E, b-carotene and phenolic compounds that may prevent LPO by scavenging free radicals. Oxidative markers were examined in brain, liver, lung, kidney, heart and spleen tissues. Compared with mice exposed to mycotoxin alone, animals co-treated with pumpkin seed oil showed lower levels of MDA. Mycotoxin exposure attenuated the activity of antioxidant enzymes; CAT activity was increased, while SOD and GPx activity were decreased. However, the administration of pumpkin seed oil to animals restored the activity of these enzymes to control levels. This study showed that treatment with pumpkin seed oil reduced the damage that could potentially be attributed to its antioxidant properties [111]. The next oil that showed protective effects in mycotoxin exposure was evening primrose oil. Kanbur et al. (2011) investigated the effect of evening primrose oil (1.5 mL/kg b.w./d by oral route) on mycotoxin toxicity in mice. After 14 days of exposure and the sacrifice of the animals, tissues (brain, liver, lung, kidney, heart and spleen) were collected and oxidative stress markers such as MDA levels and SOD, CAT and GPx activity were measured. The results showed increased MDA levels and unbalanced antioxidant enzyme activity [112]. Evening primrose oil administration positively modulated the oxidative state of mouse tissue, which is related to the level of gamma-linolenic acid. Therefore, evening primrose oil can be used in cases of mycotoxin exposure and for the prevention of poisoning with these compounds.

In addition to pumpkin seed oil, researchers have also used pumpkin seed extract to mitigate mycotoxin exposure. Alonso-Garrido et al. investigated the effects of BEA, ENN, OTA and ZEA on the in vitro BBB model. The results showed that pumpkin seed extract reduced the levels of pro-inflammatory metabolites, suggesting a protective effect against oxidative damage and inflammation induced by these mycotoxins, particularly in the context of BBB integrity and inflammatory responses [113,114].

Studies conducted on SH-SY5Y cells showed a protective effect of QUE against DON toxicity. The results showed that cells treated with QUE (1 mM) significantly reduced DON (100 mM)-induced ROS generation, LPO, MMP loss, DNA damage and cell cycle arrest and decreased the expression of neuronal biomarkers. Studies showed that QUE reduced DON-induced stress by reducing ROS production and LPO generation, maintaining MMP and DNA integrity and regulating the expression of neuronal biomarker genes in SH-SY5Y cells [74,76].

Betulinic acid (BA) demonstrates neuroprotective properties following exposure to mycotoxins. Studies have shown that BA has excellent protective properties against T-2 toxin neurotoxicity. Treatment of mice with different doses of BA (0.25, 0.5 and 1.00 mg/kg body weight) reduced cerebral hemorrhage and petechiae, improved mitochondrial morphology, enriched the number of organelles and inhibited cell apoptosis in the brain exposed to T-2 toxin (4 mg/kg). Furthermore, BA inhibited the mRNA expression of proinflammatory cytokines such as IL-1b, IL-6 and TNF-α, and increased the mRNA expression of IL-10 in the brain induced by T-2 toxin. Therefore, BA may have potential applications in the treatment of mycotoxin-induced poisoning [119]. The presented substances require further pharmacokinetic and pharmacodynamic studies. Despite their low bioavailability, they reach concentrations in tissues, mainly in the brain, that mitigate the toxic effects of mycotoxins. It should be noted that it is necessary to develop new formulations of these compounds to enhance their pharmacological effects [120,121].

## 3. Conclusions

The literature data clearly indicate that many *Fusarium* mycotoxins exhibit high neurotoxicity. The mechanism of action on the nervous system is related to increased oxidative stress, which leads to higher levels of proinflammatory cytokines, enzymatic activity and impaired neurotransmitter function. The ameliorative effects of mycotoxin neurotoxicity are mainly related to the reduction of oxidative stress using natural substances. In addition, the multi-directional effects of these substances have been shown to ameliorate other effects that are exacerbated by mycotoxin exposure. Further preclinical and clinical studies are needed to continue the search for solutions to mitigate and prevent mycotoxin-induced neurotoxicity.

## 4. Methodology

In March 2024, an extensive manual search of major electronic databases (PubMed, Google Scholar, EMBASE and Web of Science) was conducted to identify relevant articles published neurotoxicology of mycotoxins. The articles were limited to those published in English and Polish. Many search terms were used, including “mycotoxins”, “mycotoxins and human health”, “mycotoxins and neurological diseases”, “mycotoxins and neuroprotection”, “zearalenone”, “α-zearalenone”, “β-zearalenone”, “deoxynivalenol”, “toxin T-2”, “HT-2”, “beauvericin”, “enniatin A”, “enniatin B”, “mycofolenic acid” and “moniliformin”. The keywords used to obtain data on the neurotoxic effects of mycotoxins, especially *Fusarium* toxins on humans, included “mycotoxins* neurotoxicity”, “zearalenone*neurotoxicity”, “α-zearalenone*neurotoxicity”, “β-zearalenone*neurotoxicity”, “deoxynivalenol*neurotoxicity”, “toxin T-2*neurotoxicity”, “HT-2*neurotoxicity”, “beauvericin*neurotoxicity”, “enniatin A*neurotoxicity”, “enniatin B*neurotoxicity”, “mycofolenic acid*neurotoxicity”, “moniliformin*neurotoxicity” and “mycotoxins*neuroprotection”. The articles were analyzed first by title, then by abstract, and finally, by the full text. All the selected articles were the most relevant ones available for this review. The data from the time frame of 2001–2023 were used to search for information on mycotoxins and neurotoxicity, and data from 2011–2024 were used to search for information on neuroprotection. A total of 121 articles were found.

## Figures and Tables

**Table 1:** Mycotoxins produced by individual fungal species.

Mycotoxin	Fungi
AFB1	*Aspergillus flavus, A. parasiticus, A. bombycycis, A. pseudotamari, A. ochraceoroseus, A. nomius*
Fumonisin	*Fusarium verticilliodes, F. proliferatum, F. nygamai, Alternaria alternata*, *Fumonisin* spp. *lycopersici*
OTA	*Aspergillus ochraceus, A. niger, A. carbonarius, Penicillium verrucosum*
ZEA	*Fusarium culmorum, F. graminearum, F.crookwellense, F. roseum*
DON T-2, HT-2	*Fusarium graminearum, F. culmorum,* *F. sporotrichioides, F. langsethiae, F. acuminatum, F. poae*
BEA	*Fusarium* spp., *Beauveria bassiana, Isaria* spp
ENN	*Fusarium* spp., *F. oxysporum, F. avenaceum*
MPA	*Penicillium roqueforti, P. stoloniferum, P. brevicompactum, P. echinulatum*
MON	*Fusarium* spp: *Fusarium avenaceum*, *F. subglutinans*, *F. proliferatum*
STG	*Aspergillus nidulans, A. versicolor*
CPZ	*Penicillium cyclopium, P. griseofulvum, P. camemberti*, *P. commune, A. flavus, A. versicolor*

**Table 2:** The neurotoxic effects of ZEA, α-ZEL and β-ZEL.

	Effects	References
ZEA	abnormal synthesis of enzymes and neuronal factors in neurons,	
	increases oxidative stress reactions,	[29,31,33,35,37,38,40,42,43,44,45,46,47,48,49]
	induced the generation of ROS	
α-ZEL	decrease in the activity of GPx and CAT enzymes high activity of SOD and CAT increased the activity of GST increase in the population of late apoptotic cells	[50,51,52]
β-ZEL	decrease in the activity of GPx and CAT enzymes high activity of SOD and CAT increased the activity of GST arrest or delay in the G2/M and S phases activation of cell proliferation in the G0/G1 phase increase in the population of early apoptotic cells	[50,51,52]

## Data Availability

No new data were created or analyzed in this study. Data sharing is not applicable to this article.
